# Cognitive Testing Practices for Basic Military Training in the Nordic Countries: A Scoping Review

**DOI:** 10.1111/sjop.70122

**Published:** 2026-06-08

**Authors:** Emelie Wahlkrantz, Jeanette Melin

**Affiliations:** ^1^ Department of Leadership and Command & Control Swedish Defence University Karlstad Sweden

**Keywords:** conscription, general mental ability, military selection, validity

## Abstract

The Nordic countries have a long tradition of using general mental ability (GMA) tests as part of their military conscription process, a practice that began in the mid‐20th century. These Nordic tests were developed with a dual purpose—to filter out individuals unfit for military service and to rank individuals' GMA for military roles—and they share common features, including historical roots, administrative procedures, and interpretations. Initially designed exclusively for men, these tests are now taken by both men and women, reflecting shifts in societal norms. However, limited reporting on the current psychometric properties of these tests raises questions about their reliability and validity. Without up‐to‐date knowledge about the measurement characteristics of these assessments, findings derived from such tests should generally be interpreted with caution. The need for updated validation is further underscored by the extensive use of Nordic GMA test data in broader social and health research, including the Flynn effect. Such efforts will not only sustain the scientific credibility of these tests, but also ensure that data informing other research, and conclusions drawn there, are based on accurate interpretations of the GMA tests.

## Keypoints

- The Nordic countries have used GMA tests in military conscription since the mid‐20th century and have a lot in common, such as their original intention, administrative procedures, and use of stanine distribution to interpret the measurements.
- There is limited information about the psychometric properties of the GMA tests for military conscription in the Nordic countries; without up‐to‐date knowledge about the measurement quality, findings derived from such tests should generally be interpreted with caution.

## 1 Background

General Mental Ability (GMA) testing has a longstanding tradition in military selection processes and is recognized for its simple administrative process, cost‐effectiveness, and predictive validity regarding individual performance. In the Nordic countries, GMA tests have been a cornerstone of military conscription selection since the end of World War II and have been used to screen large cohorts of individuals—mostly male—for basic military training.

GMA, often referred to as intelligence, general cognitive abilities, or mental capacity, encompasses the ability to learn, adapt to new situations, and process novel information (Jensen 1980). It also includes reasoning, planning, problem‐solving, abstract thinking, and the capacity to handle complexity and learn from experience (Gottfredson 1997). GMA is typically understood as a multidimensional, hierarchical construct, with general ability at the apex and more specific abilities—such as verbal, spatial, and logical reasoning—underlying it (McGrew 2009; Salgado 2017). As a latent trait, GMA is not directly observable or measurable. Instead, it must be measured through indicators that reflect these underlying abilities (Schmidt and Hunter 2004) and often by the use of multiple choice (MC) items that test those specific abilities. Research consistently demonstrates that measurements obtained from various GMA tests strongly predict performance in both work and educational contexts (Kuncel et al. 2004; Salgado 2017; Schmidt and Hunter 1998, 2004). As a result, GMA tests are widely regarded as valid assessment tools. GMA has also proven to be a strong predictor of performance in a military context (Duckworth et al. 2019), likely due to the importance of effective decision making, adaptability, perseverance, and problem solving required in such a setting. However, the GMA tests used for military purposes are subject to strict confidentiality requirements and are not publicly available.

The origins of military GMA testing can be traced back to World War I, when the United States Army implemented the first large‐scale intelligence testing programs, such as the Army Alpha and Beta tests (Husén and Henricson 1951; Sjöberg and Wolgers 2014). These early tests laid the groundwork for the widespread use of assessments in the military, particularly in the United States and the United Kingdom. Inspired by these developments, the Nordic countries subsequently adopted similar approaches, tailoring their tests to meet the specific demands of their conscription‐based military systems.

Conscription, also known as mandatory military service or the draft, is a system in which individuals are required by law to serve in a nation's armed forces for a specified period. Historically implemented to bolster military capacity, conscription has been a key component of national defense strategies in many countries, including the Nordic countries. Given its impact on personal freedoms, it is crucial that selection processes for conscription are both valid and reliable. Ensuring fairness and accuracy in these assessments is not only a practical necessity, but also an ethical imperative, given the stakes involved in enforcing military service.

### 1.1 Review Objective

The Swedish Enlistment Battery (SEB) has recently undergone updates and modernizations. As a part of that, interest in reviewing existing knowledge in the field emerged and a literature review was conducted to assess existing knowledge of the measurement of cognitive abilities for basic military training. The aim of the scoping review was to gauge existing knowledge of testing GMA for military purposes in countries comparable to Sweden. Specifically, we focused on the Nordic countries, Norway, Denmark, and Finland (Iceland was not included as they do not have armed forces). The following research questions guided the scoping review: (i) What is included in the Nordic countries' tests of GMA for the basic military training? (ii) Which psychometric properties are reported from these tests and how do the tests perform in relation to these properties? and (iii) Are there any reports of changes in GMA from these tests?

## 2 Materials and Methods

A scoping review (Colquhoun et al. 2014; Peters et al. 2015) was undertaken to investigate the study's objective. We searched the following three databases in September 2024: Web of Science, PsychInfo, and Scopus. Searches were carried out for Denmark, Finland, Norway, and Sweden; these were varied slightly depending on the number of hits for each country. For example, when doing searches for Sweden, an additional search block was added, with the name of the test in question, as the number of hits was quite high when using only the three search blocks used for the other three countries (see Appendix 1 for a detailed declaration). We limited our inclusion criteria to academically relevant content such as reports, peer‐reviewed articles, dissertations, and textbooks, excluding materials such as news articles or blog posts. Acceptable languages included English, Swedish, Norwegian, or Danish, whereas Finnish could not be included due to its lack of mutual intelligibility with the other Nordic languages understood by the authors. In January 2024, before the search blocks were finalized, attempts were made to contact earlier collaborators working with these tests in Denmark, Norway, and Finland, asking for published materials on their respective tests to capture relevant articles and map out adequate search words. However, at the time we did not reach Finland, but followed up during the fall of 2024. In all responses, test confidentiality was cited as the key reason for limited reporting on the tests.

The strategy also included manual searches of reference lists in studies selected from the electronic search and looking for articles that have subsequently referenced key studies from the electronic search. The electronic database searches identified 646 records, with three additional records found in manual searches. This amounted to a total of 649 records after duplicates were removed. The online software Rayyan was used to collect and review titles and abstracts.

In the selection of studies to be included in the review, inclusion and exclusion criteria were used as a guide. Grounds for inclusion were (1) the study should focus on testing of GMA in a military context, (2) the sample should be potential recruits/conscripts or military personnel, (3) the aim of the study must be to assess the properties of the GMA test or GMA in this group. Exclusion criteria were (1) studies utilizing results from GMA testing to study something else, such as health outcomes, (2) studies assessing other types of cognitive abilities, such as emotional control.

Titles and abstracts were screened regarding the inclusion and exclusion criteria, and if the study aims were met. As a result, 609 records were rejected, leaving 40 records. These were assessed in full text and an additional four articles were then excluded for not meeting inclusion criteria. Three papers, all from the 1940s, were not available in full text and could not be retrieved. This process resulted in the inclusion of 33 papers, see Figure 1. See also Appendix 2 for a full list of references included per country.

**FIGURE 1:**
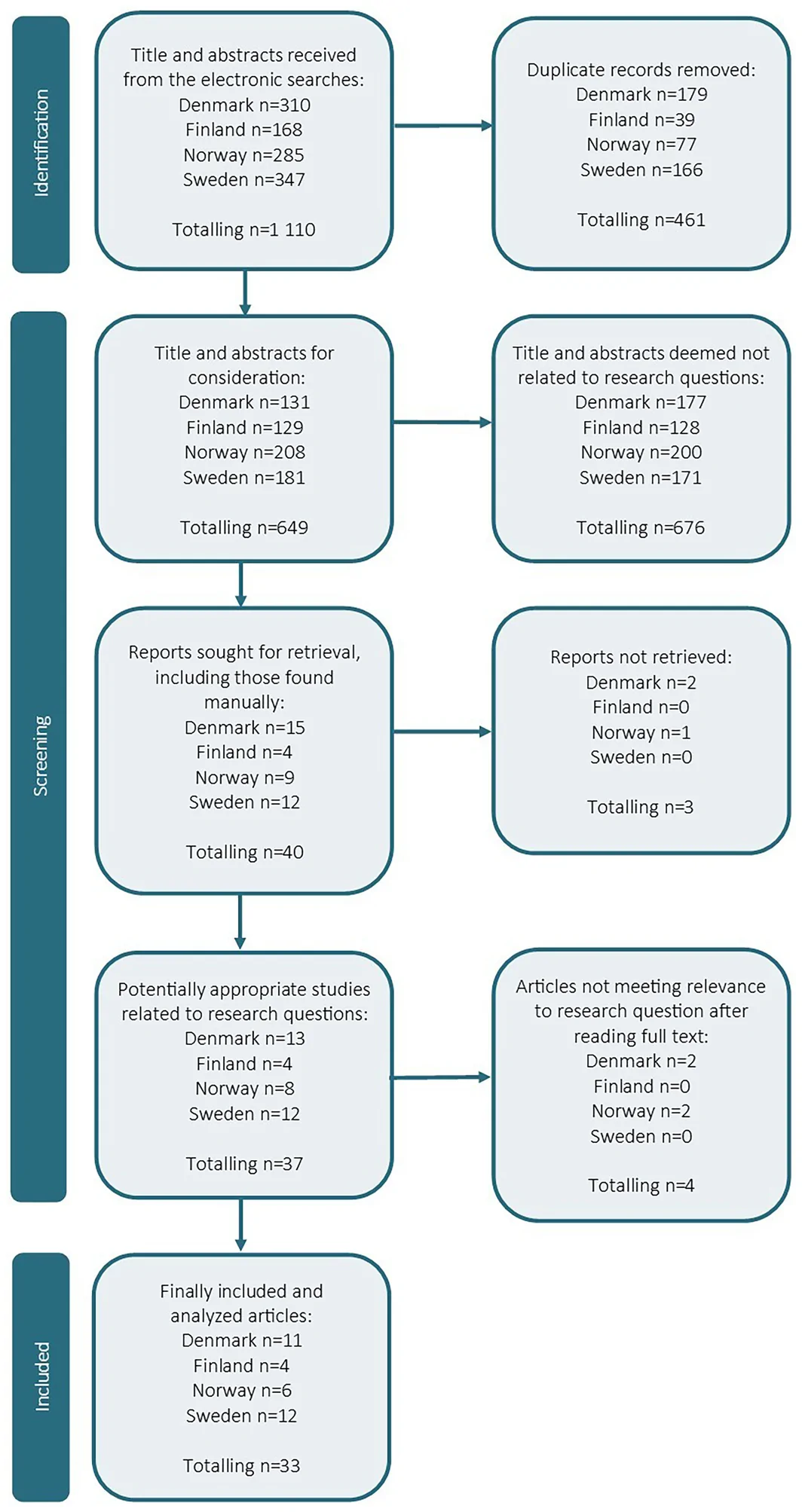
The review process.

## 3 Results

### 3.1 Theoretical Underpinnings and Practical Orientation of the Tests

In the Nordic countries, cognitive testing for military conscription has been standard since the 1940s and 1950s. The overarching aim of the tests is to evaluate cognitive abilities independently of educational background, ensuring that potential recruits/conscripts possess essential mental capacities for military service. These tests draw on a dual foundation. Firstly, they were developed for practical military selection needs to assess the vast majority of males in each age cohort, inspired by American group testing methods from the World Wars (Carlstedt 2000). Secondly, they are grounded in psychological theories of general mental abilities. See Table 1 for an overview of the differences and similarities between the Nordic tests.

**TABLE 1:** Overview of GMA test implementations and structure in the Nordic countries.

	Name of test in native language	Used since	Theoretical construct	Subtests	Time limit	Test theory	Measurement	Interpretations	Administration	Conscription
Denmark	Børge Prien's Prøve (BPP)	1957	GMA^a^	4 subtests: RPM^e^, verbal test, numerical test and visuospatial test	Time limit per subtest; total 45 mins.	IRT^f^	Sum score	Stanine	Computerized in group sessions	Only men are conscripted. Women participate voluntarily
Finland	Peruskoe‐1	1955, latest revision 1981	GMA	3 subtests: verbal, arithmetic and visuospatial (like RPM^e^)	Time limit per subtest; total 37 mins.	CTT^g^	Sum score	Stanine	Pen and paper in group sessions	Only men are conscripted. Women participate voluntarily
Norway		1954	GMA	3 subtests: figure matrices (like RPM^e^), synonyms and arithmetic tests.	Time limit per subtest; total 53 mins.	CTT^g^	Sum score transformed to percentiles	Stanine	Computerized in group sessions	Gender neutral conscription
Sweden	I‐prov	1944, latest revision 2025	GMA, Gf^b^, Gc^c^ and Gv^d^	6 subtests: number series, paper fold and cut, synonyms, antonyms, paper folding, dice	Time limit per subtest: total 70 mins.	IRT^f^	Theta value^h^	Stanine	Computerized in group sessions	Gender neutral conscription

^a^ General Mental Ability (GMA).

^b^ Fluid intelligence.

^c^ Crystallized intelligence (Gc).

^d^ Visuospatial intelligence (Gv).

^e^ Ravens Progressive Matrices (RPM).

^f^ Item Response Theory (IRT).

^g^ Classical Test Theory (CTT).

^h^ An estimate of a person's ability expressed on a logit scale.

Historically, these tests targeted young men, although Sweden (Jonsson et al. 2024) and Norway (Køber et al. 2017) have recently expanded this to also include women. Nowadays, roughly 5% of each age cohort undergo conscription in Sweden (Jonsson et al. 2024), a number that is expected to increase in the coming years. Over the years, the Norwegian military has reduced the size of its conscript pool, which now encompasses around 15% of each age cohort completing service (Køber et al. 2017). In 2013, Dutton and Lynn reported that most Finnish men over the age of 18 (around 88%) complete military service. However, later reporting says around 33% (Jonsson et al. 2024). In Denmark, among those eligible for conscription, a lottery (sic!) is held to select individuals for conscription, resulting in around 20%–25% of the male population being selected for conscription in each cohort (Teasdale et al. 2011).

In Denmark, cognitive testing for conscripts began in 1957, focusing primarily on fluid intelligence. The test is named after its creator Børge Prien,^1^ Børge Priens Prøve (BPP), and was modeled after Raven's Progressive Matrices. The Danish test sought to measure verbal, numerical, and spatial abilities, similar to Thurstone's primary mental abilities (Teasdale 2009) and remains largely unchanged since its introduction (Nielsen et al. 2020). In Finland, cognitive tests, first administered in 1955, are designed to measure general intelligence (Leikas et al. 2009). Since 1981, the test has been known as Peruskoe‐1 or the Basic test. Norway introduced its military cognitive test in 1954, and it is largely unchanged (Køber et al. 2017), with only minor alterations—the arithmetic subtest was modernized in the mid‐1960s and converted to a multiple choice format in the 1990s (Fadum et al. 2022).

In Sweden, cognitive testing for conscription began in 1944 and has undergone multiple revisions, with updates in 1947, 1948, 1949, 1954, 1959, 1967, 1980, 1994, 2000, and 2025. Initially influenced by Spearman's general ability concept, Swedish testing evolved in the 1950s to reflect Thurstone's model of primary mental abilities, which shaped test design for over three decades (Carlstedt 2000). In the 1960s, an increased interest in technical knowledge and ability emerged to meet the requirements of new mechanical and technical jobs in the Swedish military. This was reflected in the addition of subtests in technical comprehension. From the 1990s onwards, tests were strongly guided by modern theoretical and methodological development with its roots in Scandinavia (ibid). In international contexts, the term SEB is commonly used, while in Swedish it is referred to as I‐prov. Given the number of updates, the year of launch is typically added; for example, the former version was SEB‐2000, and the current one is SEB‐2025. The SEB‐2025 was designed to theoretically align with the dominant intelligence theory, emphasizing GMA and three broad cognitive abilities: fluid intelligence (Gf), crystallized intelligence (Gc), and visuospatial intelligence (Gv) (Melin, Wahlkrantz, et al. 2025).

### 3.2 The Nordic GMA Tests and Subtests

The Nordic countries employ various subtests to assess GMA for military purposes. In Denmark, the BPP includes four subtests to assess GMA: Letter matrices, evaluating pattern recognition; Verbal Analogies, testing verbal reasoning, Number Series, assessing numerical reasoning; and Geometric Figures, measuring spatial reasoning (Nielsen et al. 2020; Teasdale et al. 2011). Finland's Peruskoe‐1 has three subtests: Verbal, focusing on vocabulary and relationships between words; Arithmetic, testing skills through number sequences and basic math problems, and Visuospatial, similar to Raven's Matrices, where test takers complete patterns by identifying the missing piece (Tiihonen et al. 2005). In Norway, the military test has three subtests: Arithmetic, assessing mathematical skills and logical reasoning, Word similarity, evaluating vocabulary through synonym selection, and Figures, a nonverbal test similar to Raven's Matrices (Fadum et al. 2022; Skoglund et al. 2014; Sundet et al. 2004). Sweden's SEB‐2025 includes six subtests; two subtests per broad cognitive ability (Melin, Wahlkrantz, et al. 2025). This structure was designed to reduce the impact of subtest‐specific variance and to ensure sufficient breadth for adequate construct representation. The subtests included are Number Series and Paper fold and cut for assessing Gf, Synonyms and Antonyms for assessing Gv, and Paper foldning and Dice for assessing Gv.

### 3.3 Administration

The administration of GMA tests for military selection is computerized in all Nordic countries, except Finland, where optical response forms are used. The Danish test was computerized in 2010–2011 (Nielsen et al. 2020; Teasdale et al. 2011) and the Norwegian test was computerized in 2011 (Køber et al. 2017). The SEB was computerized in the 1990s (Mårdberg and Carlstedt 1998). All Nordic countries administer the tests in proctored group contexts. Side note: Norway is currently exploring the possibility of unproctored testing in a home environment (Norrøne 2023).

All countries apply time restrictions per subtest, which gives Denmark a total time limit of 45 min for all subtests (Teasdale 2009), Finland a total time of 37 min, Norway a total of 53 min (Fadum et al. 2022), and Sweden a total time of 70 min.

MC item seems to be the conventional type of question in the GMA tests utilized in Finland (Dutton and Lynn 2013), Norway (Skoglund et al. 2014), and Sweden (Mårdberg and Carlstedt 1998). In the 1950s, in Sweden, the choice of MC items was justified by greater objectivity when scoring test takers' answers because differences in writing ability were eliminated. It was also justified by time saved in application and scoring, as tests were completed with paper and pencil (Husén and Henricson 1951). Denmark is the exception from the traditional MC item format, utilizing 100 response options to minimize the potential effect of guessing, a design choice by its creator, Børge Prien (Teasdale 2009). Items are presented in order of increasing difficulty per subtest in all Nordic countries (Fadum et al. 2022; Lönnqvist et al. 2008; Nielsen et al. 2020).

### 3.4 Obtaining Measurements

In Denmark, scores on the BPP are calculated by summing the raw scores across its four subtests, counting only the number of correct answers achieved within the time limit. The maximum score is 78, reflecting the total number of items, with a minimum cutoff score of 28 to be considered for conscription (Teasdale et al. 2011). Similarly, in Finland, scores from the 120 items are totaled (Leikas et al. 2009). In Norway, the scores from each subtest are transformed into normally distributed F‐scores and summed to yield a combined measure of GMA (Fadum et al. 2022). In Sweden, the SEB‐2025 is designed with parallel test forms. Correct responses in each subtest are equated across forms using the Leunbach model and transformed into a logit scale according to the Rasch model, yielding theta values (i.e., estimates of a person's abilities expressed on a logit scale) for each of the six subtests. The average of the six theta values is then used as the measure of GMA.

For all four countries, the measurements are then transformed into standard nine (Stanine) categories (Fadum et al. 2022; Leikas et al. 2009; Teasdale 2009) used for the selection to the basic military training. Stanine categories are based on the standard deviation of a normal distribution, with 4%, 7%, 12%, 17%, 20%, 17%, 12%, 7%, and 4% of test takers per category respectively. That means Stanine 4–6 comprises 54% of test takers with average abilities, Stanine 1–3 is below average and 7–9 above average. There is no clear justification in the literature for the specific use of stanines. In Sweden, however, their application can be traced back to the 1950s, when the Swedish Armed Forces introduced them as a way to summarize results from various tests and assessments conducted during military conscription (Annell 2025).

### 3.5 Psychometric Properties of the Tests

In Denmark, the four subtests generated a reliability coefficient (Cronbach's alpha), ranging from 0.72 to 0.82 (Teasdale et al. 2011). Mortensen et al. (1989) have shown that the BPP correlates highly (0.82) with the Wechsler Adult Intelligence Scale, concluding that the BPP has a strong general intelligence (g) component. Likewise, Teasdale's (2009) validation of the BPP against educational level has shown a consistent correlation of 0.55 over the years, with Geometrical Figures demonstrating slightly lower correlation than the other three subtests. Furthermore, Hartmann and Teasdale (2004) compared high and low ability test takers and found that the Danish test was a better indicator for GMA among high ability test takers than low ability test takers. Despite its age, Teasdale et al. (2011) suggest that the BPP remains valid due to its abstract nature, which has kept it relevant over time. In 2020, Nielsen et al. published research where they evaluated the four subtests of the BPP using the Leunbach model and found support for the sum score being used. However, it is worth noting that they did not investigate the test at the item level. Historically, ceiling effects have been minimal in the Danish test; prior to 1998, fewer than 10% of individuals answered more than 85% of the items correctly (Teasdale and Owen 2008). Additionally, Teasdale et al. (2011) investigated motivation among test takers and found that individuals with lower motivation generally perform better on the test than those with higher motivation, which indicates that low motivation does not undermine results in the BPP.

In Finland, the test–retest reliability for the subtests has been reported to range from 0.76 to 0.88 (Sinivuo 2003, as referenced in Tiihonen et al. 2005). Unfortunately, no other studies of the psychometrical properties of the Peruoske‐1 were found in our scoping review.

In Norway, Sundet et al. (1998, as referenced in Skoglund et al. 2014) examined the test–retest reliability of the Norwegian test and found it to be 0.84, 0.72, and 0.90 respectively for the three subtests. Additionally, Skoglund et al. (2014) performed a correlation study comparing the Norwegian recruitment test to the Wechsler Adult Intelligence Scale. This yielded a correlation of 0.72, which the researchers conclude indicates support for the construct validity of the Norwegian test. Recently, the measurement properties of the matrices test were examined by Helland‐Riise et al. (2024) through a unidimensional IRT model, and it was found to fit well in all groups and years tested. However, the authors report that the test's precision was found to be poor at the upper end of the scale, with a possible ceiling effect. At the item level, some difference in functioning between males and females was also found, but at the test level it was deemed to function equally well for males and females. Even so, the authors encourage caution in interpretations from previous studies including the GMA test.

Since the latest version of SEB has only been in use for a few months, no studies on the full SEB‐2025 are yet available. However, a recent publication on the psychometrics of one of the try‐out subtests demonstrated that the PFC test is a robust and unbiased tool for assessing logical ability among conscripts (Wahlkrantz and Melin 2025). In the coming years, a full‐scale evaluation of SEB‐2025 is planned, along with studies of its construct and predictive validity. Moreover, a number of earlier publications have examined the psychometrics of SEB‐2000. For instance, solid construct validity, including correlations with the Swedish Scholastic Aptitude Test (Carlstedt and Gustafsson 2005) and rates of return to schooling (Nordin 2008), has been demonstrated. Research has also highlighted important psychometric aspects from SEB 2000, such as the role of item sequencing, the impact of time limits on visuospatial strategies, and the predictive value of word frequency in verbal items (Carlstedt et al. 2000; Ullstadius et al. 2002, 2004). Earlier versions—SEB‐80 and SEB‐94—showed strong G‐factors, though with some variation in Gc and Gv loadings (Carlstedt and Mårdberg 1993; Mårdberg and Carlstedt 1998).

### 3.6 Changes in GMA

Research across the Nordic countries reveals both positive and negative Flynn effect trends in cognitive abilities. In Denmark, Teasdale and Owen (1989) documented a significant Flynn effect from the 1950s to the 1980s, particularly among lower percentiles, but saw a decline starting in the early 2000s, suggesting a negative Flynn effect (Teasdale and Owen 2005). This is assumed to be connected to the egalitarian Danish educational system focusing on students with lower abilities (Teasdale and Owen 1989). Similarly, Hegelund et al. (2021) identified GMA gains between the 1940s and 1980s in Danish conscript data, while the 1990s showed stagnation or, in some cases, deflation of GMA. In Finland, Dutton and Lynn (2013) noted gains from 1988 to 1997, with subsequent declines, potentially explained by dysgenic fertility,^2^ according to the authors. Norwegian data shows similar trends, with Flynn effect gains until the mid‐1990s when a sharp decline in arithmetic and vocabulary skills began (Sundet et al. 2004). Storsve et al. (2018) attributed this not to immigration, but to social and technological shifts, such as a reliance on digital tools for calculations and outdated vocabulary tests. In addition, a recent study of temporal trends and measurement invariance in the Norwegian Armed Forces suggests that temporal variations in intelligence scores may be more accurately attributed to shifts in test properties and cognitive specialization (Nordmo et al. 2025). However, attributing observed declines in intelligence test scores to issues of measurement invariance does not rule out the possibility that actual declines in cognitive abilities have also occurred. In Sweden, from the 1970s to the beginning of the 1990s, visuospatial scores increased significantly, while verbal tests stagnated, indicating varied trends across different GMA abilities (Rönnlund et al. 2013).

## 4 Discussion

The Nordic countries have had a long‐standing tradition of GMA tests for military conscription since the mid‐20th century. These tests were originally designed for men but nowadays are also applied to women. These tests reflect unique historical and methodological choices because they were developed with a dual purpose: as a practical tool for group testing to sift out individuals unsuitable for conscription; and for ranking individuals' GMA for different military purposes. The Nordic tests have a lot in common, such as their original intention, administrative procedures, and use of stanine distribution to interpret the measurements. They also display some differences, such as the Norwegian, Finnish, and Danish tests remaining largely unchanged since their introduction, whereas the Swedish test has undergone multiple revisions over the years. Moreover, the Danish test (Nielsen et al. 2020) and the recent versions of the Swedish test were reportedly developed through modern test theory, while the Finnish and Norwegian are based on classical test theory. However, recent work on the Norwegian test is also based in this tradition, utilizing IRT methods (Helland‐Riise et al. 2024).

There is limited information about the psychometric properties of the GMA tests for military conscription in the Nordic countries, which raises important questions about their validity and reliability. Without up‐to‐date knowledge about the measurement characteristics of these assessments, findings derived from such tests should generally be interpreted with caution. Similar concerns have been expressed in Norwegian research, which highlights the need for more careful interpretation of GMA data gathered from these conscription assessments, “previous studies using the Norwegian GMA data must be interpreted with more caution than has been the case so far in the field of intelligence” (Helland‐Riise et al. 2024, 16) and “suggest that the interpretations of such trends [Flynn effect] might be confounded by both changes in cognitive abilities and shifts in test characteristics” (Nordmo et al. 2025, 8). One potential reason for the limited reporting on these tests could be the confidentiality requirements surrounding military selection processes. However, disclosure of details of the test content or specific items is not strictly necessary. Instead, properties of the test and its items can be reported without compromising test security, as recently demonstrated by Helland‐Riise et al. (2024) and Wahlkrantz and Melin (2025). Another possible explanation is the prevailing notion within psychology that once a test is validated, it remains valid indefinitely. However, such a view is overly naïve, as test properties may change over time and item drift can challenge earlier conclusions about validity (Chan et al. 1999; Melin, Wahlkrantza, et al. 2025; Shiu et al. 2013). While there are no established standards for how frequently test properties should be re‐evaluated, we argue that regular reassessment is essential to ensure the continued quality and validity of the test.

The GMA tests in this study serve not only as tools for selecting individuals for conscription—a high stakes decision with potentially life altering consequences, particularly in wartime—but also as general indicators of GMA, informing many other research areas. It is, therefore, of the utmost importance to ensure that these tests have strong measurement properties, are monitored over time, and remain fair to all test takers. The Swedish Defense Conscription and Assessment Agency have taken steps to ensure that the new SEB‐2025 is robust. The authors are also aware of similar work being conducted in Norway with the Norwegian test (c.f., Norrøne and Nordmo 2025). We emphasize the importance of publishing empirical findings on these tests to enhance transparency and facilitate informed interpretations by researchers and practitioners alike.

The implications of the little knowledge about psychometric properties are considerable, given the extensive use of Nordic cognitive conscription test data in broader social and health research. Numerous studies have used the results of these measurements of GMA to investigate various societal and health‐related issues. Examples include family studies based on GMA, such as father‐son comparisons (Björklund et al. 2010), twin comparisons (Nilsen et al. 1984), sibling comparisons (Sundet et al. 2008), and birth order comparisons (Bjerkedal et al. 2007). GMA has also been compared to other psychological constructs, such as emotional stability (Leikas et al. 2009), resilience (Friborg et al. 2005), and personality (Lönnqvist et al. 2008). Additionally, it has been used in health studies related to birth circumstances (Eide et al. 2005; Eriksen et al. 2010); alcohol consumption (Rogne et al. 2021); psychiatric disorders (Vassend et al. 1994); mental and physical impairments (Gravseth et al. 2008); suicide (Gravseth et al. 2010); and aging (Poranen‐Clark et al. 2016). The results from these studies can potentially be used to inform public policy and healthcare practices, underscoring the importance of accurate measurement tools as unverified or outdated tests may lead to misinterpretations and incorrect prioritizations.

Another important aspect relates to fairness and equity in testing, as raised by Helland‐Riise et al. (2024). These tests are developed and standardized for a male population. However, nowadays they are also applied to female test takers; in Finland and Denmark on a voluntary basis, but in Norway and Sweden through conscription. There are also demographic changes to consider, for example changes in the proportion of test takers with an immigrant background. Therefore, thorough investigation of the measurement properties of tests is warranted, to ensure that they function similarly for all subgroups, and that tests maintain their quality over time, so that all test takers get a fair estimate of their GMA, and that test results are comparable across years.

In the present review, we deliberately chose to limit the scope to the Nordic countries, acknowledging the unique shared context of these countries. At the same time, we recognize that a broader European or even global perspective would be of great interest to a wider audience. However, given the substantial challenges we encountered in gathering comprehensive information even within the Nordic region, we believe that a more extensive review would likely require an international collaboration to ensure both breadth and depth of coverage.

## 5 Conclusions and Implications for Future Research and Practice

Moving forward, further research into and publications about the psychometric properties of such assessments is essential to support their continued use in military selection processes and in broader research contexts that use results from GMA tests. Such efforts will not only sustain the scientific credibility of these tests but also ensure that data informing other research and conclusions drawn there are based on accurate interpretations of the GMA tests.

## Author Contributions

Conceptualization, Methodology: J.M. and E.W. Formal analysis, Investigation, Writing – original draft: E.W. Writing – review and editing, Supervision: J.M. All authors read and approved the final version of the manuscript.

## Funding

This work was supported by the Swedish Defence Conscription and Assessment Agency.

## Data Availability

The data that supports the findings of this study are available in the appendices.

## Endnotes

^1^
Prien's father in law was the Danish statistician and mathematician George Rasch, who was also consulted in the creation of BPP (Nielsen et al. 2020).

^2^
Dysgenic fertility refers to a pattern where individuals with lower GMA have higher reproductive rates than those with high GMA, potentially lowering the average GMA in future generations.

## Appendix 1

**Table:**

	Block 1	Block 2	Block 3	Block 4
Denmark	Denmark OR Danish	“cognitive abilit*” OR “mental ability*” OR intellig* OR IQ OR aptitude	conscript* OR military* OR “armed force*” OR army OR enlist*	
Finland	Finland OR Finnish	“cognitive abilit*” OR “mental ability*” OR intellig* OR IQ OR aptitude	conscript* OR military* OR “armed force*” OR army OR enlist*	
Norway	Norway OR Norwegian	“cognitive abilit*” OR “mental ability*” OR intellig* OR IQ OR aptitude	conscript* OR military* OR “armed force*” OR army OR enlist*	
Sweden	Sweden OR Swedish	“cognitive abilit*” OR “mental ability*” OR intellig* OR IQ OR aptitude	conscript* OR military* OR “armed force*” OR army OR enlist*	I‐prov OR Iprov OR “Swedish enlistment battery” OR SEB OR measur*
